# Two cases of lobectomy for lung cancer after transcatheter aortic valve implantation

**DOI:** 10.1186/s40792-018-0548-7

**Published:** 2018-12-03

**Authors:** Hideki Nagata, Ryu Kanzaki, Takashi Kanou, Naoko Ose, Soichiro Funaki, Yasushi Shintani, Masato Minami, Isamu Mizote, Yasushi Sakata, Koichi Maeda, Toru Kuratani, Yoshiki Sawa, Meinoshin Okumura

**Affiliations:** 1 Department of General Thoracic Surgery, Osaka University Graduate School of Medicine, L5-2-2 Yamadaoka, Suita, Osaka 565-0871 Japan; 2 Department of Cardiology, Osaka University Graduate School of Medicine, Suita, Japan; 3 Department of Cardiovascular Surgery, Osaka University Graduate School of Medicine, Suita, Japan; 4 Department of General Thoracic Surgery, National Hospital Organization Toneyama Hospital, Toyonaka, Japan

**Keywords:** Lung cancer, Lobectomy, TAVI

## Abstract

**Background:**

The age of patients with lung cancer is advancing, and the number of patients with lung cancer who have cardiac diseases is expected to increase. Recently, the rate of transcatheter aortic valve implantation (TAVI) has increased as treatment for aortic stenosis (AS). TAVI is minimally invasive compared with conventional aortic valve replacement. We herein report two patients with lung cancer who underwent lobectomy after TAVI for severe AS.

**Case presentation:**

Two patients with AS and lung cancer were treated with two-stage surgery of TAVI followed by lobectomy. In patient 1 (77 years of age), conventional aortic valve replacement was considered to be risky because of his history of coronary artery disease and thoracic aortic aneurysm and his relatively high logistic euroSCORE. He underwent TAVI followed by right middle and lower lobectomy. In patient 2 (75 years of age), TAVI was chosen because the patient had poor ADL due to spinal canal stenosis and had taken immunosuppressant agents after a kidney transplantation. He underwent TAVI followed by right lower lobectomy. The postoperative course of the two patients was uneventful.

**Conclusions:**

Two-stage surgery of TAVI and lung resection could be a viable option for patients with both lung cancer and severe AS, for whom conventional AVR by an open-heart operation is not indicated.

## Background

The age of patients with lung cancer is advancing, and the number of patients with lung cancer who have cardiac diseases is expected to increase. Recently, the rate of transcatheter aortic valve implantation (TAVI) has increased as treatment for aortic stenosis (AS). TAVI is minimally invasive compared with conventional aortic valve replacement. We herein report two patients with lung cancer who underwent lobectomy after TAVI for severe AS. These patients were not indicated for conventional aortic valve replacement (AVR) by open-heart operation due to their high-risk status with this surgery.

## Case presentation

### Case 1

**Fig. 1:**
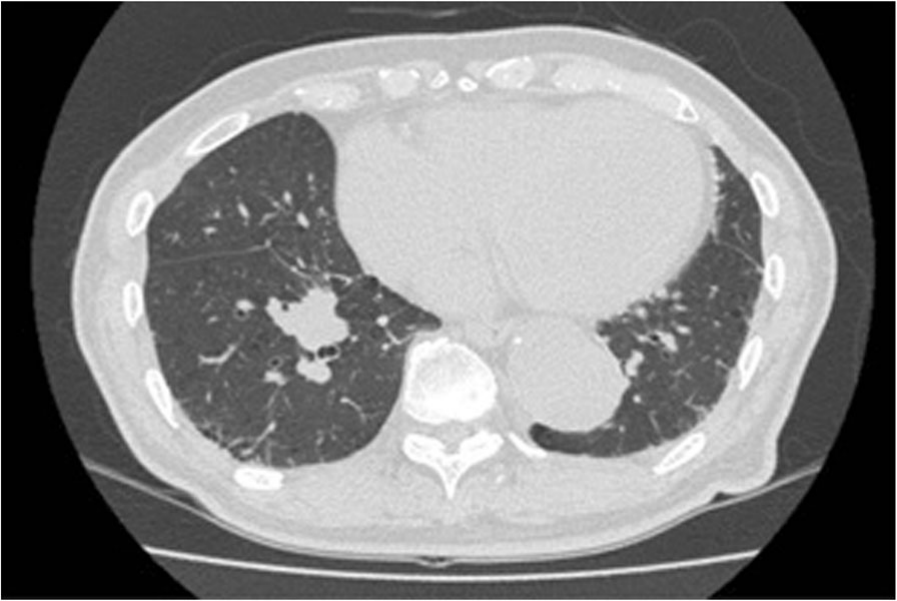
Axial image of a chest computed tomography (CT) scan

A 77-year-old man was referred to our department for the evaluation of a pulmonary nodule in the right lower lobe detected by chest computed tomography (CT). He had a surgical history of thoracic endovascular aortic repair and coronary-artery bypass. The pulmonary nodule had increased in size, so lung cancer was suspected (Fig. 1). The clinical stage was determined to be IA-3 by radiologic examinations. He was also diagnosed with AS simultaneously.

**Fig. 2:**
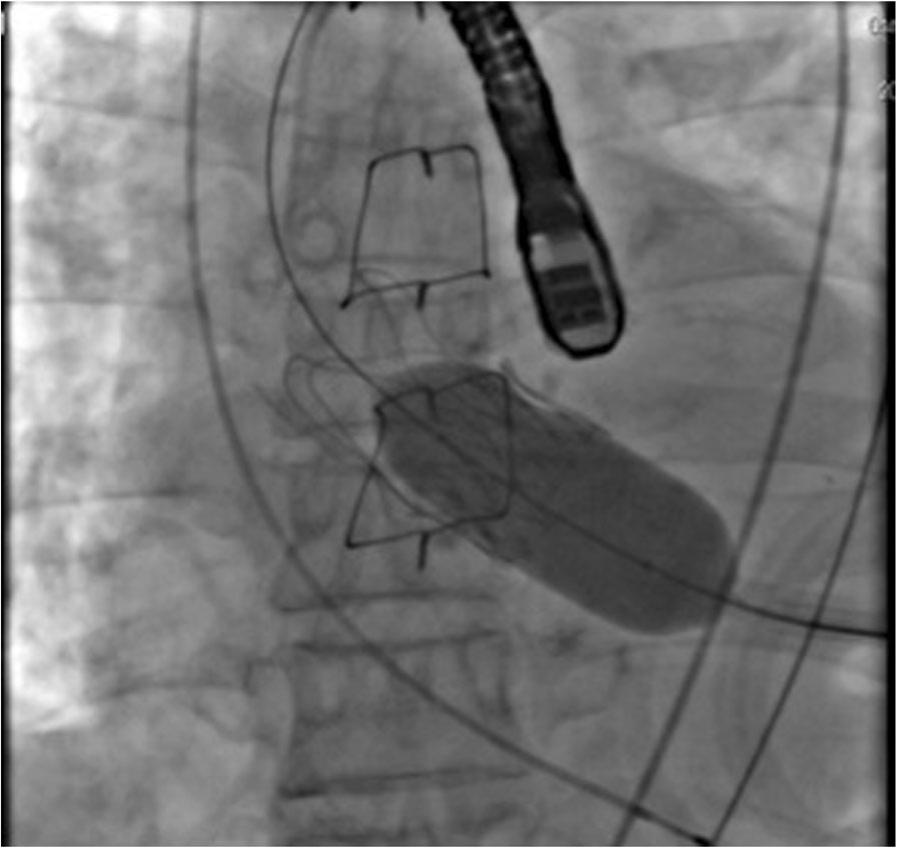
Deployment of the artificial valve at TAVI

Because his AS was severe (mPG 44 mmHg, AVA 0.73 cm^2^) and there was a possibility of sudden cardiac death, treatment for AS was considered to be mandatory before pulmonary resection. In addition to his history of coronary artery disease and thoracic aortic aneurysm, his logistic euroSCORE was relatively high (39.8%). Based on these data, conventional AVR was considered to be risky. TAVI was therefore selected for AS, and a trans-apical TAVI with left-sided intercostal thoracotomy was successfully performed without postoperative complications (Fig. 2).

We performed surgery for lung cancer 70 days after TAVI. Perioperative heparin bridging was performed for the low-dose aspirin therapy he had been taking. Right middle and lower lobectomy with mediastinal lymph node dissection was performed. We initially tried to perform video-assisted thoracic surgery (VATS); however, severe adhesion was present in the right chest cavity due to the effects of coronary artery bypass grafting, which he had undergone for coronary artery disease. We therefore performed thoracotomy. The pathologic diagnosis was squamous carcinoma, pT1cN0M0 stage IA3. The postoperative course was uneventful. Fourteen months after surgery, the patient is doing well without relapse or cardiac symptoms.

### Case 2

A 75-year-old man with a history of chronic renal failure, hypertension, atherosclerosis obliterans, and spinal canal stenosis was referred to our hospital for the treatment of AS. He had undergone living-donor kidney transplantation 13 years earlier and had taken immunosuppressant agents. Moreover, before receiving kidney transplantation, he had been on hemodialysis for 15 years. TAVI was selected to treat his severe AS because of his medical history as described above.

**Fig. 3:**
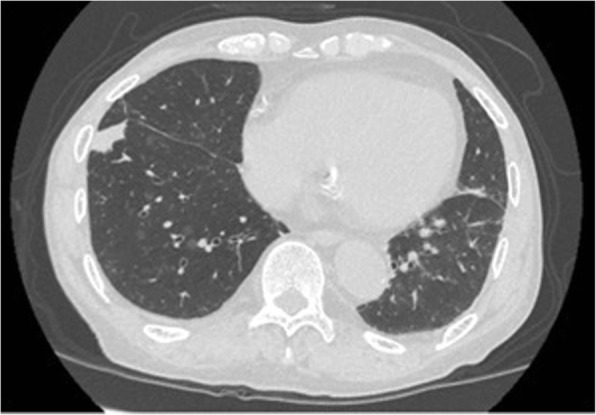
Chest CT scan before TAVI

**Fig. 4:**
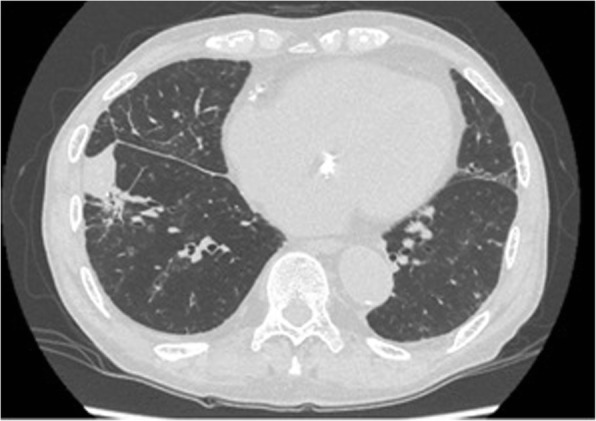
Chest CT scan 8 months after TAVI. The nodule in the right lower lobe grew larger

At the preoperative examination for AS, CT showed an abnormal shadow in the right lower lobe (Fig. 3). At this time, an inflammatory nodule was suspected, so we planned to follow this abnormal shadow by CT. TAVI was performed through a retrograde transfemoral approach successfully without postoperative complications. The shadow in the right lower lobe enlarged, and lung cancer was suspected (Fig. 4). A transbronchial lung biopsy was performed, and the pathologic examination revealed adenocarcinoma. The clinical stage was stage IB, so we planned surgery.

We performed right lower lobectomy 9 months after TAVI. Perioperative heparin bridging was performed for the low-dose aspirin therapy he had been taking. Thoracotomy was needed due to the presence of severe diffuse adhesion of the entire surface of the lung and right chest cavity, probably due to pleuritis. Right lower lobectomy was performed without complications. The pathologic diagnosis was squamous carcinoma, pT1bN0M0 stage IB. The postoperative course was uneventful. Eight months after surgery, he is doing well without recurrence.

## Discussion

Recently, the age of patients with lung cancer has been advancing, and the number of patients afflicted with both cardiac disease and malignancy is increasing [1–4]. Older people are known to have a higher frequency of AS, so we expect the number of patients with lung cancer and AS to increase in the future [5].

The conventional treatment for AS is open-heart surgery under median sternotomy with extracorporeal circulation. As a less-invasive treatment method for AS, TAVI with AVR performed through a catheter without open-heart surgery has become widely used. Neither extracorporeal circulation nor stopping the heart beating is required for TAVI, making this approach minimally invasive compared with conventional AVR.

TAVI is indicated for patients who would be at a high risk with open-heart operation. A high logistic euroSCORE (> 20%) and Society of Thoracic Surgeons score (> 10%) are good indicators of such patients [6]. In addition to these scores, the presence of comorbidities should be considered. However, the long-term outcomes of TAVI are still unclear, and the durability of the artificial valve used in TAVI is considered to be approximately 5 to 7 years. Therefore, the age of the patients is important to consider when TAVI is performed [5]. In the present two cases, TAVI was indicated because the patients were in their late 70s and were considered high-risk patients for conventional AVR.

Whether to perform concomitant or two-stage procedures for patients with lung cancer and cardiac disease simultaneously is controversial [3]. Generally, two-stage surgery is performed because concomitant surgery of the heart and lung is highly invasive and is associated with an increased risk of complications after surgery [3].

However, if the time interval between the first and second surgery is expected to affect the progression of the lung cancer, concomitant surgery should be considered, or the second surgery should be performed without delay.

There have been a few cases of lung resection after TAVI reported in the English literature [7–9]. Drevet et al. [7] firstly reported the case of a 75-year-old patient with AS and lung cancer who underwent left upper sleeve lobectomy 72 h after TAVI. This patient suffered from pneumonia and atrial fibrillation in the perioperative period, but he is free from tumor recurrence at 1 year after lobectomy. In our cases, the time interval between TAVI and lobectomy was relatively long at 70 days and 9 months, due to the patients’ wishes and follow-up of the size of the tumor. Bauer et al. [10] reported that, 24 h after TAVI, a significant reduction in the transaortic mean pressure gradient was achieved, and the left ventricular ejection fraction increased. However, the recovery of the cardiac function after the open-heart operation is known to be delayed because of oxidative stress and apoptosis of cardiomyocytes evoked by inflammation induced by the surgery [11]. The time interval between the first and second surgery can therefore be reduced with TAVI. Furthermore, TAVI may be particularly beneficial for cancer patients, as this procedure does not require extracorporeal circulation. The use of extracorporeal circulation for cancer patients may encourage the progression of cancer through the dissemination of cancer cells [12]. Based on these advantages of TAVI, it can be important to decide the approximate date of the second surgery for cancer after TAVI in advance.

In conclusion, pulmonary resection followed by TAVI can be safely performed, and this strategy could be a viable option for patients with both lung cancer and severe AS, for whom conventional AVR by an open-heart operation is not indicated.

## Abbreviations

AS: Aortic stenosis
AVR: Aortic valve replacement
CT: Computed tomography
TAVI: Transcatheter aortic valve implantation
